# Chronic Metabolic Acidosis Activates Renal Tubular Sodium Chloride Cotransporter through Angiotension II-dependent WNK4-SPAK Phosphorylation Pathway

**DOI:** 10.1038/srep18360

**Published:** 2016-01-05

**Authors:** Yu-Wei Fang, Sung-Sen Yang, Chih-Jen Cheng, Min-Hua Tseng, Hui-Min Hsu, Shih-Hua Lin

**Affiliations:** 1 Division of Nephrology, Department of Medicine, Shin Kong Wu Ho-Su Memorial Hospital, Taipei, Taiwan; 2 Graduate Institute of Medical Science, National Defense Medical Center, Taipei, Taiwan; 3 Division of Nephrology, Department of Medicine, Tri-Service General Hospital, Taipei, Taiwan; 4 Division of Pediatric Nephrology, Chang Gung Memorial Hospital, Taoyuan, Taiwan

## Abstract

The mechanism by which chronic metabolic acidosis (CMA) regulates sodium (Na^+^)-chloride (Cl^−^) cotransporter (NCC) in the renal distal convoluted tubules remains unexplored. We examined the role of STE20/SPS1-related proline/alanine-rich kinase (SPAK) and with-no-lysine kinase 4 (WNK4) on expression of NCC in mouse models of CMA. CMA was induced by NH_4_Cl in wild type mice (WTA mice), SPAK, and WNK4 knockout mice. The quantities of Ncc mRNA, expression of total NCC, phosphorylated (p)-NCC, SPAK and WNK4 in the kidneys as well as NCC inhibition with hydrochlorothiazide and Na^+^ balance were evaluated. Relative to WT mice, WTA mice had similar levels of Ncc mRNA, but increased expression of total and p-NCC, SPAK, and WNK4 and an exaggerated response to hydrochlorothiazide which could not be observed in SPAK or WNK4 knockout mice with CMA. In WTA mice, increased plasma renin activity, aldosterone and angiotensin II concentrations accompanied by a significantly negative Na^+^ balance. High Na^+^ diet abolished the enhanced NCC expression in WTA mice. Furthermore, an angiotensin II type 1 receptor blocker rather than a mineralocorticoid receptor antagonist exerted a marked inhibition on Na^+^ reabsorption and NCC phosphorylation in WTA mice. CMA increases WNK4-SPAK-dependent NCC phosphorylation and appears to be secondary to previous natriuresis with volume-dependent angiotensin II activation.

The kidney has a major role in maintaining systemic acid-base homeostasis. Accumulation of acids or depletion of bases in the body can lead to chronic metabolic acidosis (CMA). In response to CMA, both proximal and distal renal tubules have their distinct roles in adaptation. In proximal renal tubules, most of filtered bicarbonate (HCO_3_^−^) are reabsorbed and this process is initiated by the electroneutral Na^+^-H^+^ exchanger 3 (NHE3) in the luminal membrane and the kidney-type electrogenic Na^+^/HCO_3_^−^ cotransporter (NBC) in the basolateral membrane^1^,^2^. As for distal renal tubules, new HCO_3_^−^ are regenerated to replenish the consumptive HCO_3_^−^ via the vacuolar-type H^+^-ATPases in the luminal membrane and anion exchanger 1 in the basolateral membrane^3^,^4^. Any defects in the reabsorption and regeneration of HCO_3_^−^ cause proximal and distal renal tubular acidosis, respectively^5^.

Besides renal tubular acid-base adaptation, renal salt handling is also significantly influenced by CMA. The presence of natriuresis has been reported in normal healthy subjects with NH_4_Cl-induced CMA and also in acid-loaded rats^6^,^7^. This natriuresis may be related to the reduced sodium (Na^+^)-chloride (Cl^−^) reabsorption in proximal renal tubules as shown in micropuncture study in the dogs with NH_4_Cl-induced CMA^8^. Despite the natriuresis, the expression and activities of Na^+^-dependent transporters along renal tubules could be significantly changed in CMA. For example, the upregulation of type 3 Na^+^/H^+^ exchanger (NHE3) in proximal tubules, Na^+^-K^+^-2Cl^−^ cotransporter (NKCC2) in medullary thick ascending limbs and epithelial Na^+^ channel (ENaC) in the collecting ducts^9^,^10^,^11^. With respect to distal convoluted tubules (DCTs), Na^+^-Cl^−^ cotransporter (NCC) responsible for 5% to 7% of the fine-tuning reabsorption of filtered Na^+^ has been reported to be changed in acidosis. Acute metabolic acidosis alters NCC activities associated with the downregulation of NCC density and reduction in protein abundance^12^,^13^. However, a recent study showed that the amount of NCC expression seemed to be associated with the time course of CMA which remained unchanged at early phase but significantly increased thereafter^11^. Nevertheless, the mechanism of NCC expression and activity in CMA remains unclear.

Recent studies have clearly demonstrated that NCC expression and activities can be regulated by two upstream kinase, STE20/SPS1-related proline alanine-rich kinase (SPAK) and with-no-lysine kinase 4 (WNK4)^14^,^15^. SPAK phosphorylates NCC on residues T53, T58, and S71^16^,^17^. In addition, SPAK null mice recapitulate the phenotype of Gitelman syndrome (GS), inactivating mutations in *SLC12A3* gene encoding NCC, with markedly diminished total and phosphorylated (p)-NCC expression^16^. The WNK4, as upstream of SPAK, can also activate NCC by phosphorylating SPAK^18^. WNK4 knockout mice exhibit Gitelman-like syndrome with significantly reduced expression of SPAK and NCC^19^. Thus, the WNK4-SPAK signaling appears to be a major pathway to mediate NCC activation. Hormones such as aldosterone, angiotensin II, insulin, and vasopressin have been shown to regulate this signal pathway^20^.

In this study, we investigated whether NCC was activated and regulated through WNK4-SPAK pathway in CMA induced by exogenous NH_4_Cl administration in different mice including wild type (WT), SPAK, and WNK4 knockout mice. The results to be reported indicated that mice with CMA exhibited the NCC activation through WNK4-SPAK-dependent pathway mediated by salt depletion with an enhanced angiotensin II.

## Results

### Blood and urine biochemistry of WT and WTA mice

Blood and urine samples were collected from WT mice and WT mice receiving NH_4_Cl (WTA) for 5 days (Table 1). Compared to WT mice, WTA mice exhibited significantly decreased blood pH and HCO_3_^−^ concentrations with preserved renal function (Table 1). WTA mice also had significantly higher plasma renin activity (PRA), angiotensin II and aldosterone concentrations. In urine, pH and citrate excretion were significantly lower but calcium excretion was significantly higher in WTA mice than WT mice.

### Expression of NCC and p-NCC of WT and WTA mice

**Figure 1:**
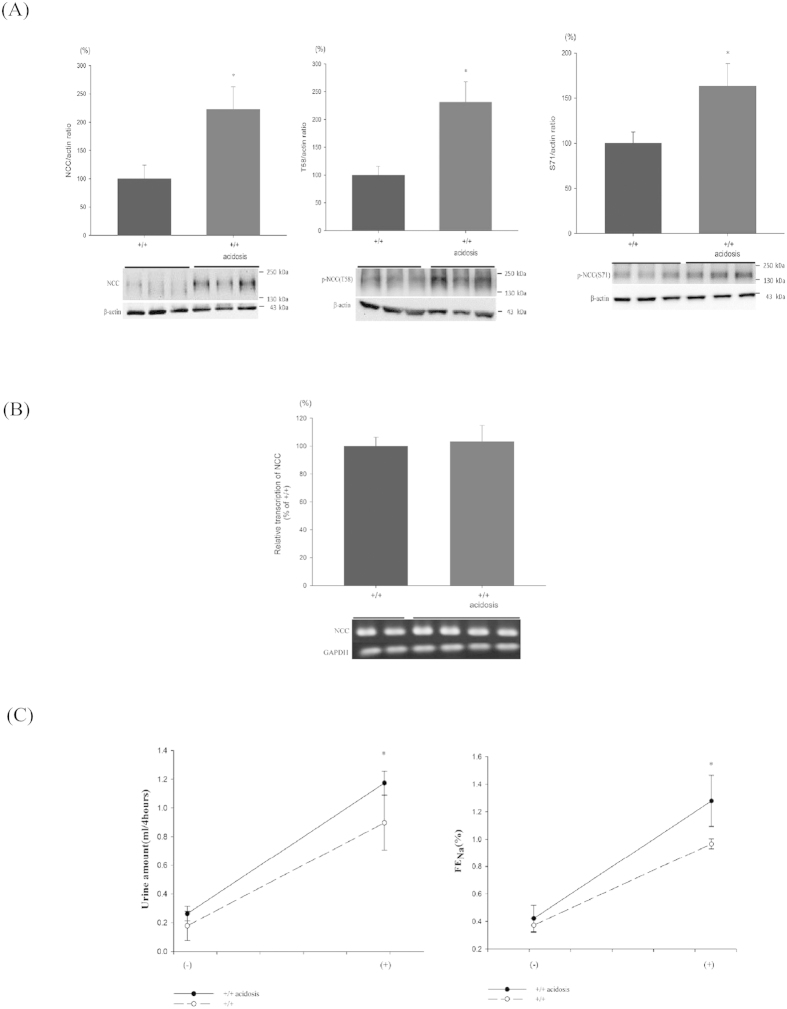
Expression of NCC and effect of hydrochlorothiazide (HCTZ) in WT and WTA mice. (**A**) Semiquantitative immunoblotting of total kidney extracts indicate that total and p-NCC (T58 and S71) were significantly enhanced in WTA mice (6 mice/group, 3 separate gels). The blots and gels were cropped and the full-length blots/gels are presented in Supplementary Fig. S1. (**B**) Reverse transcriptase PCR and real-time PCR indicated similar levels of NCC transcripts in WTA and WT mice (4 mice per group). The blots and gels were cropped and the full-length blots/gels are presented in Supplementary Fig. S2. (**C**) Following a single dose of HCTZ, WTA mice produced more urine and had higher FeNa (8 mice per group). *p < 0.05 *vs.* WT mice.

To assess NCC expression and phosphorylation in CMA, the relative levels of total and p-NCC as well as Ncc transcripts in the kidney tissues were measured (Fig. 1). Relative to WT mice, the renal tissues of WTA mice had 2.2-fold increased in NCC, 1.6-fold in p-NCC(T58), and 2.3-fold in p-NCC(S71) (Fig. 1A). The amounts of Ncc mRNA transcripts remained unchanged between WT and WTA mice (Fig. 1B).

### Response to hydrochlorothiazide in WT and WTA mice

Since both total and p-NCC were significantly increased in WTA mice, hydrochlorothiazide (HCTZ, 25 mg/kg) was administered to determine whether the NCC function was increased. The resulting urine output and fractional excretion of Na^+^ (FENa) were used as an index of the NCC function in response to its inhibitors. The amounts of both urine and FENa were markedly increased after a single dose of HCTZ in WTA mice, suggesting that the NCC function was enhanced (Fig. 1C).

### Expression of SPAK and WNK4 in WT and WTA mice

**Figure 2:**
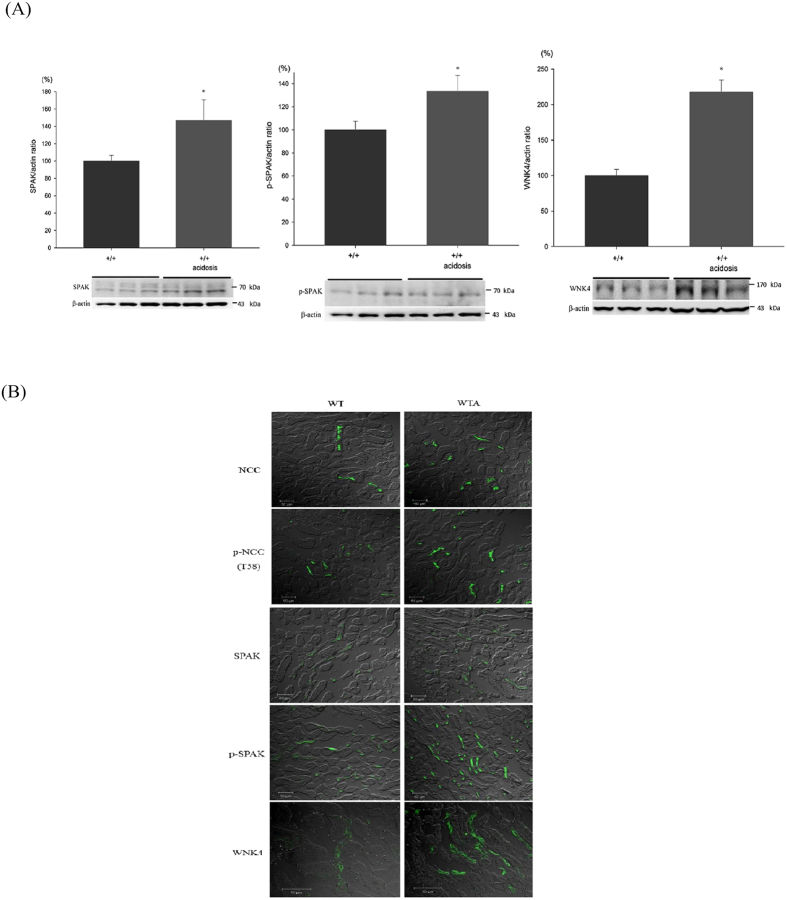
Expression of SPAK and WNK4 in WT and WTA mice. (**A**) Semiquantitative immunoblotting of total kidney extracts indicated increased expression of total SPAK, p-SPAK, and WNK4 in WTA mice (6 mice/group, 3 separate gels). The blots and gels were cropped and the full-length blots/gels are presented in Supplementary Fig. S3. (**B**) Immunofluorescence staining of NCC and p-NCC in the DCTs indicated total and p-NCC were more luminally condensed with greater expression of SPAK and WNK4 in WTA mice. *p < 0.05 *vs.* WT mice.

NCC expression, phosphorylation, and activity were all significantly increased in WTA mice. Thus, we examined the expression of SPAK and WNK4 to identify their roles. WTA mice had 1.3- to 1.4-fold higher levels of SPAK and p-SPAK and a 2.1-fold higher level of WNK4 than WT mice (Fig. 2A). Moreover, the cellular distributions of total and p-NCC in the DCT were more luminally condensed in parallel with increased SPAK and WNK4 expression in WTA mice (Fig. 2B).

### NCC phosphorylation in SPAK^−/−^ mice with NH_4_Cl-induced CMA

**Figure 3:**
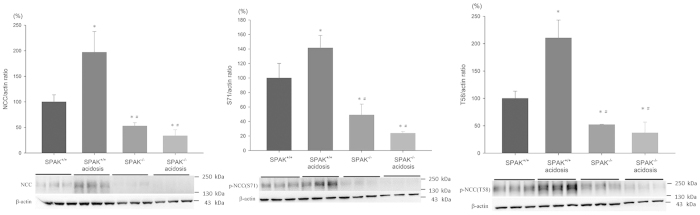
Effect of acidosis on expression and phosphorylation of NCC in SPAK^−/−^ mice. SPAK knockout mice had reduced levels of total and p-NCC in kidney tissues. Acidosis increased the expression of total and p-NCC in SPAK^+/+^ mice, but not in SPAK^−/−^ mice (6 mice/group, 3 separate gels). *p < 0.05 *vs.* SPAK^+/+^ mice; ^#^*p* < 0.05 *vs.* SPAK^+/+^ mice with acidosis. The blots and gels were cropped and the full-length blots/gels are presented in Supplementary Fig. S4.

The enhanced expression of total and p-NCC in WTA mice may be mediated by a SPAK-dependent or independent pathway. We examined the expression of total and p-NCC in SPAK^−/−^ and SPAK^+/+^ mice with and without CMA (Fig. 3). Diminished expression of total and p-NCC were present in the SPAK^−/−^ mice (approximately 0.5-fold). Although CMA may have 2-fold and 1.5-fold increase in total and p-NCC in the SPAK^+/+^ mice but not in the SPAK^−/−^ mice (reduced to 2/3-fold, Fig. 3). These results indicated that increased NCC expression and phosphorylation in CMA were mainly through SPAK-dependent pathway.

### NCC phosphorylation in WNK4^−/−^ mice with NH_4_Cl-induced CMA

**Figure 4:**
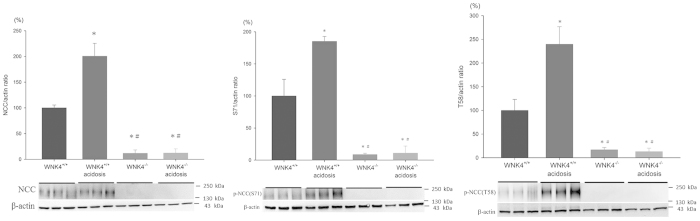
Effect of acidosis on expression and phosphorylation of NCC in WNK4^−/−^ mice. WNK4 knockout mice had reduced levels of total NCC and p-NCC in kidney tissues. Acidosis increased the expression of total and p-NCC in WNK4^+/+^ mice, but not in WNK4^−/−^ mice (6 mice/group, 3 separate gels). *p < 0.05 *vs.* WNK4^+/+^ mice; ^#^*p* < 0.05 *vs.* WNK4^+/+^ mice with acidosis. The blots and gels were cropped and the full-length blots/gels are presented in Supplementary Fig. S5.

Similarly, the enhanced total and p-SPAK expression may be due to WNK4-dependent or independent pathway. We examined NCC expression under the condition of WNK4 deletion *in vivo* and induced with CMA. The expression of total and p-NCC were markedly diminished in the WNK4^−/−^ mice (approximately 0.1-fold). In contrast to the WNK4^+/+^ mice which had 1.5 to 2.5-fold increase in total and p-NCC, CMA failed to increase NCC expression and phosphorylation in WNK4^−/−^ mice suggesting that increased SPAK-(p)NCC expression in CMA was mainly mediated by WNK4 (Fig. 4).

### Na^+^ balance and NCC expression in CMA

**Figure 5:**
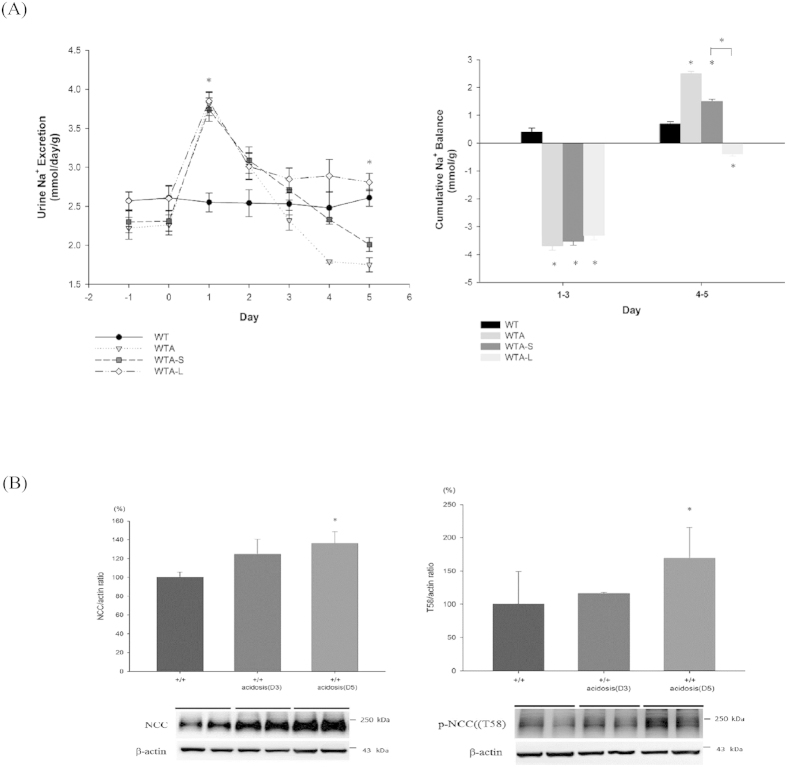
Effect of acidosis on the Na^+^ balance and expression of NCC. (**A**) WTA mice experienced early natriuresis, but this abated after day 5 (upper panel), leading to a negative Na^+^ balance before day 3 and a positive balance thereafter (lower panel). WTA mice given losartan (WTA-L mice) had high urinary Na^+^ and a negative Na^+^ balance on day 5, and this differed significantly from WTA mice given spironolactone (WTA-S mice) (6 mice/group). (**B**) At day 3, the levels of total and p-NCC were similar between WT and WTA mice, but WTA mice had significantly higher levels of NCC expression on day 5 (6 mice/group, 2 separate gels). *p < 0.05 *vs.* WT mice; ^#^*p* < 0.05 *vs.* WTA-S mice. The blots and gels were cropped and the full-length blots/gels are presented in Supplementary Fig. S6.

Since enhanced expression of WNK4, SPAK and NCC were involved in WTA, it remained to be further clarified whether it was regulated by either acidosis per se or changes in volume status. Due to the limitations of an *in vivo* study for examining the direct effects of acidosis, we recorded daily urinary Na^+^ excretion to determine the influence of volume status (Fig. 5). Significant natriuresis (WTA mice: 3.81 ± 0.15; WT mice: 2.55 ± 0.12 mmol/day/g; *p* < 0.05) started at 1 day after NH_4_Cl loading and continued for 2 days, causing a negative Na^+^ balance before day 3. On day 4 to 5, the urine Na^+^ excretion gradually decreased (WTA mice on day 5: 1.75 ± 0.09; WT mice on day 5: 2.61 ± 0.11 mmol/day/g; *p* < 0.05), with a compensated positive Na^+^ balance thereafter (Fig. 5A). WT and WTA mice had similar expression of total NCC and p-NCC on day 3, but WTA mice had significantly increased levels on day 5, in parallel with their changes in Na^+^ excretion (Fig. 5B).

### Effects of high Na^+^ diet on NCC expression in WTA mice

**Figure 6:**
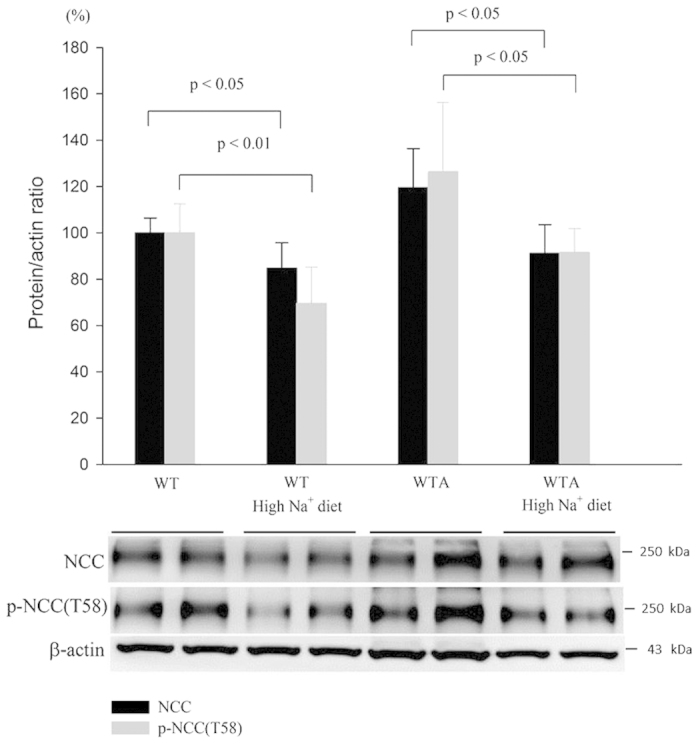
Expression of total and p-NCC in WTA mice with high Na^+^ diet. Semiquantitative immunoblotting of total kidney extracts indicated that the levels of total and p-NCC were significantly increased in WTA mice, but could be attenuated by high Na^+^ diet. (4 mice/group, 2 separate gels). The blots and gels were cropped and the full-length blots/gels are presented in Supplementary Fig. S7.

Increased Na^+^ excretion and a negative Na^+^ balance occurred in the early phase of CMA, so we determined the effect of this early natriuresis on NCC activation by administering normal or high Na^+^ diet to WT and WTA mice. In WT mice, high Na^+^ diet reduced the expression of total and p-NCC by about 20–25%. As for WTA mice, high Na^+^ diet attenuated the increased expression of total and p-NCC by approximately 10–15%. This result suggested that high Na^+^ intake may prevent the volume-depleted NCC activation in CMA (Fig. 6).

### Effects of RAA inhibitors on salt balance and NCC expression in WTA mice

**Figure 7:**
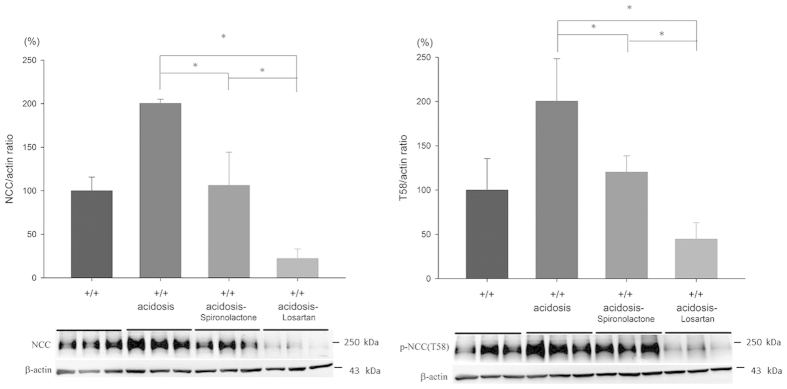
Effects of spironolactone and losartan on expression of NCC in WTA mice. WTA mice had increased expression of total and p-NCC. Losartan significantly attenuated this effect whereas spironolactone had lesser degree of attenuation (6 mice/group, 3 separate gels). *p < 0.05 *vs.* WTA mice; ^#^*p* < 0.05 *vs.* WTA-S mice. The blots and gels were cropped and the full-length blots/gels are presented in Supplementary Fig. S8.

High Na^+^ intake can prevent activation of NCC in CMA, but it remains to be determined whether the volume-dependent activation of the renin-angiotensin-aldosterone system (RAAS) is involved in this response. Since both angiotensin II and aldosterone are well-known to stimulate NCC expression, its individual role was evaluated by administering an angiotensin II type 1 receptor (AT1R) blocker (losartan) and a mineralocorticoid receptor (MR) antagonist (spironolactone) in WTA mice (abbreviated as WTA-L and WTA-S mice, respectively). In WTA-S mice, the amounts of urinary Na^+^ excretion on day 5 was slightly higher than WTA mice (2.01 ± 0.09 mmol/day/g) causing a similar positive Na^+^ balance. On the other hand, administration of losartan can maintain higher Na^+^ excretion on day 5 (2.81 ± 0.11 mmol/day/g), leading to a more negative Na^+^ balance (Fig. 5A). The enhanced total and p-NCC expression in CMA could be significantly abrogated by losartan (approximately reduced to 20–25%) rather than spironolactone. These results indicated that an AT1R blocker had a predominant effect to reduce Na^+^ reabsorption and NCC phosphorylation in WTA mice (Fig. 7).

## Discussion

In this study, we demonstrated that mice with NH_4_Cl-induced CMA (WTA mice) had increased levels of total and p-NCC and an exaggerated response to thiazide diuretics. The concomitantly increased WNK4 and SPAK expression in WTA mice and the lack of NCC activation in SPAK^−/−^ or WNK4^−/−^ mice with CMA all supported that WNK4 and SPAK as upstream regulators. In WTA mice, increased PRA as well as elevated angiotensin II and aldosterone concentrations were accompanied by a significant negative Na^+^ balance and the enhanced NCC expression could be abolished by high Na^+^ diet suggestive of volume-dependent RAAS activation. An AT1R blocker rather than a MR antagonist exerted marked inhibition on Na^+^ reabsorption and NCC phosphorylation supporting that the activation of WNK4-SPAK signaling on NCC phosphorylation in CMA was mainly secondary to salt depletion with activated angiotensin II.

NCC expression and activity in DCTs has been investigated in this present study using different animal models with CMA. The results indicated that WTA mice had increased expression of total and p-NCC with unchanged Ncc transcripts suggestive of the post-translational regulation of NCC^21^. In response to thiazide diuretics as an inhibitor of NCC activity, there was an exaggerated response in WTA mice, indicative of enhanced NCC activities. Since NCC phosphorylation in represent of NCC activity was enhanced in WTA mice, we then examined the role of its upstream kinase such as WNK4-SPAK signal pathway^15^. Both SPAK and WNK4 had markedly increased expression in WTA mice, implicating this signal pathway in the activation of NCC. It is possible that other kinases also regulate NCC activity through WNK4-SPAK-independent pathways, but the lack of NCC activation in SPAK^−/−^ and WNK4^−/−^ mice supported that WNK4 and SPAK were the major upstream regulators of NCC in CMA^22^.

The activation of WNK4-SPAK-(p)NCC pathway in CMA has been shown to be regulated by hormones, such as angiotensin II, aldosterone, insulin, vasopressin, and non-hormonal factors such as sympathetic nerve stimulation^23^. The significant elevation in PRA and aldosterone levels in WTA mice pointed to the potential involvement of RAAS. With a salt balance study, we found that a negative Na^+^ balance with early natriuresis was obviously present in CMA consistent with the results of previous studies. The increased urinary Na^+^ excretion in CMA is related to the reduced filtrated HCO_3_^−^ to reabsorb Na^+^ in the proximal renal tubules. As shown in the microperfusion study, acidosis-induced natriuresis could be resulted from impaired lumen-to-blood transport of Na^+^ and HCO_3_^−^ in proximal renal tubules without changes in backflux^24^. In dog kidneys, serum HCO_3_^−^ level is inversely correlated with the degree of renal tubular Na^+^ loss, suggesting a causal relationship^25^. Accordingly, the activation of RAAS may be highly associated with a negative Na^+^ balance with volume depletion in CMA. The attenuation of the activated NCC expression in CMA with high Na^+^ diet also supported this notion.

Both angiotensin II and aldosterone are known to enhance WNK4-SPAK-(p)NCC expression and activity. Angiotensin II, a potent vasoconstrictor, can be regulated by renin secretion in response to volume depletion. Infusion of angiotensin II increases NCC expression and Na^+^ reabsorption in DCT which can’t be blocked by spironolactone^26^. This aldosterone-independent effect can be further supported in adrenalectomized rats which angiotensin II can increase SPAK and NCC phosphorylation^27^. On the other hand, incubation of angiotensin II in oocytes co-expressed WNK4 and NCC can induce NCC phosphorylation which can be abolished when losartan was added^28^,^29^,^30^. In WNK4 knockout mice, angiotensin II failed to induce SPAK and NCC phosphorylation^31^. Accordingly, these findings indicate that angiotensin II stimulates WNK4-SPAK expression and activity independent of aldosterone. Recently, angiotensin II has also been shown to decrease the ubiquitination of WNK4 through binding to protein kinase C to phosphorylate Kelch-like protein 3^32^. As for aldosterone, which can be induced by hormones, such as angiotensin II and adrenocorticotropic hormone, and non-hormone factors, such as potassium, can induce p-NCC expression through WNK4 which was also independent to angiotensin II^33^,^34^,^35^. To test the effects of angiotensin II and aldosterone apart on WNK4-SPAK-(p)NCC activation in CMA, each inhibitor was used to separate their effects on this signal pathway. Losartan, as an angiotension II inhibitor, exerted much more inhibition on Na^+^ reabsorption and reduction in NCC expression than a MR antagonist, spironolactone. Angiotensin II appeared to be the major regulator on WNK4-SPAK-(p)NCC activation in CMA with volume depletion.

Besides the increase in Na^+^ reabsorption in the proximal and distal renal tubules, the increased angiotensin II during CMA is also crucial to enhance renal acidification by participating HCO_3_^−^ reabsorption and H^+^ secretion^36^,^37^,^38^. The findings that angiotensin II to augment single nephron acidification in rats with subtotal nephrectomy and losartan exerting more degree of pH-lowering in human with CMA than spironolactone support its role in the maintenance of acid-base homeostasis^39^,^40^. During CMA, the hypovolemia-dependent angiotensin II and aldosterone activation have synergic effects on Na^+^ reabsorption by stimulating the activities of NCC in the DCTs and ENaC in the collecting ducts, respectively. The angiotensin II-dependent WNK4-SPAK-(p)NCC activation causes the decreased Na^+^ delivery to the collecting ducts and thus attenuates the effects of aldosterone on ENaC. Since angiotension II-dependent WNK4-SPAK-(p)NCC activation was present in CMA, the AT1R blocker and/or NCC inhibitors should be used with caution in such setting to avoid the exacerbated natriuresis, worsening metabolic acidosis and even hypokalemia.

In conclusion, the findings of our present study show that the increased NCC expression and activation is present in CMA which is highly associated with the enhanced WNK4-SPAK signal pathway using WNK4^−/−^ and SPAK^−/−^ mice. Previous natriuresis with volume-dependent RAAS activation rather than metabolic acidosis *per se* primarily contributed to this signal activation. Like aldosterone paradox, angiotensin II rather than aldosterone is a major regulator of the WNK4-SPAK-(p)NCC pathway in the setting of volume depletion during CMA.

## Methods

### Animals and treatment protocols

The experimental protocol was approved by the Institutional Animal Care and Use Committee of the National Defense Medical Center (Taipei, Taiwan) and all experiments were conducted in accordance with relevant guidelines. We created SPAK^−/−^ and WNK4^−/−^ mice as previously described^16^. All mice were given tap water *ad libitum*^16^. Wild type (WT), SPAK^−/−^, and WNK4^−/−^ mice (age: 6–8 months; weight: 25–30 g) were treated with 280 mM NH_4_Cl in tap water *ad libitum* to induce CMA and were fed with either normal Na^+^ diet (0.4% NaCl [w/w]) or high Na^+^ diet (4.0% NaCl [w/w]) for 5 days. Water and food intake and urine production were determined by use of metabolic cages. WTA mice were given with spironolactone (20 mg/kg/day) which was dissolved in ethanol or losartan (20 mg/kg/day) by adding to the drinking water on day 3 to 5 following NH_4_Cl administration.

### Blood and urine measurements

Blood was collected from the submandibular venous plexus under light anesthesia with ether. All mice were housed in metabolic cages for urine collection. Serum and urine biochemical analyses were performed as previously described^16^. In brief, a blood gas analyzer (ABL505; Radiometer, Copenhagen, Denmark) was used to measure pH, which was calculated as HCO_3_^−^. Urine samples were collected directly from the urinary bladders. The urine pH was measured immediately after collection with a pH microelectrode (PHR146; Lazar, Los Angeles, CA). Measurements of plasma and urine urea nitrogen, creatinine, electrolytes, and osmolality were performed with an automated device (AU 5000 Chemistry Analyzer; Olympus, Tokyo, Japan). Angiotensin II concentrations were measured by the mouse kit of enzyme-linked immunosorbent assay (ELISA; BlueGene BioTech. Co., Ltd., Shanghai, China).

### Immunoblotting and immunofluorescence

Semiquantitative immunoblotting and immunofluorescence staining were performed as previously described^16^. Proteins from whole kidney homogenates and membrane fractions (for apically expressed channels and transporters) were loaded onto SDS-PAGE gels and subjected to silver staining and immunoblotting of β-actin. The antibodies used in this study were our previously generated rabbit anti-p-NCC (T58 and S71) and anti-p-SPAK (S383)^16^, and commercially available rabbit anti-SPAK (Cell Signaling), NCC (Millipore), and anti-WNK4 (5713S; Cell Signaling) antibodies. All primary antibodies were used at 1:200 dilution for immunoblotting. Alkaline phosphatase-conjugated anti-IgG antibodies (1:3000 dilution, Promega) were used as secondary antibodies for immunoblotting. Alexa 488 or 546 dye-labeled (Molecular Probes) secondary antibodies were used for immunofluorescence staining. The immunofluorescence images were obtained from a LSM510 Meta camera that was attached to a fluorescence microscope (Carl Zeiss, Inc., Oberkochen, Germany).

### Quantitative real-time RT-PCR

Total kidney RNA was isolated by a standard reverse transcriptase (RT) protocol, and expression of Ncc was determined by the reverse transcriptase polymerase chain reaction (RT-PCR) and real-time PCR, as previously described^17^. The primers for mouse Ncc were: forward 5′-AGC CCA GCC ACT TAA CAC AC-3′ (on exon 1) and reverse 5′-GGA TCA CTC CCC AGA TGT TG-3′ (on exon 3). The primers for mouse β-actin were: forward 5′-ACC ACA CCT TCT ACA ATG AGC-3′ and reverse 5′-GGC ACA GTG TGG GTG ACC-3′.

### Hydrochlorothiaizide challenge test

Intraperitoneal hydrochlorothiazide (25 mg/kg) was administered to WT and WTA mice. Urine samples at 4 h after this treatment were collected for analysis.

### Statistical analysis

SPSS 18.0 for Windows (SPSS, Chicago, IL) was used for statistical analysis. All results are expressed as means ± standard deviations (SDs). The significance of differences between groups was examined by an unpaired Student’s *t*-test or a one-way ANOVA followed by Scheffe’s *post hoc* test. A *p*-value less than 0.05 was considered significant.

## Additional Information

**How to cite this article**: Fang, Y.-W. *et al.* Chronic Metabolic Acidosis Activates Renal Tubular Sodium Chloride Cotransporter through Angiotension II-dependent WNK4-SPAK Phosphorylation Pathway. *Sci. Rep.* **6**, 18360; doi: 10.1038/srep18360 (2016).

## Supplementary Material

Supplementary Information

## Figures and Tables

**Table 1:** Blood and urine biochemistry of wild type (WT) mice and WT mice given NH_4_Cl for induction of metabolic acidosis (WTA).

Characteristic	WT mice(n = 8)	WTA mice(n = 8)
**Blood**		
Hematocrit (%)	33 ± 1	32 ± 1
pH	7.41 ± 0.1	7.29 ± 0.03*
HCO_3_^−^ (mmol/L)	24.3 ± 0.1	15.5 ± 1.4*
Na^+^ (mEq/L)	153.3 ± 6.2	152.3 ± 3.1
K^+^ (mEq/L)	4.5 ± 0.3	4.4 ± 1.3
Cl^−^ (mEq/L)	112.7 ± 3.5	119.4 ± 2.1*
Ca^2+^ (mg/dL)	10.5 ± 0.2	10.5 ± 0.6
BUN (mg/dL)	30.3 ± 3.2	29.8 ± 2.9
Creatinine (mg/dL)	0.06 ± 0.03	0.06 ± 0.02
Aldosterone (pg/mL)	1408 ± 89.5	1735 ± 151.2*
PRA (ng/mL/hr)	9.5 ± 3.4	22.0 ± 2.9*
Angiotensin II (pg/mL)	687.8 ± 248.8	1052.7 ± 201.4*
**Urine**		
pH	7.4 ± 0.2	5.9 ± 0.3*
UUN (mg/dL)	2684 ± 197	2489 ± 325
Creatinine (mg/dL)	48.2 ± 5.5	40.6 ± 3.5*
Osmoality (mOsm/kg)	1541 ± 87	1577 ± 157
Na^+^/Creatinine (mEq/mg)	0.16 ± 0.01	0.17 ± 0.03
K^+^/Creatinine (mEq/mg)	0.45 ± 0.12	0.44 ± 0.05
Cl^−^/Creatinine (mEq/mg)	0.27 ± 0.03	0.27 ± 0.35
Ca^2+^/Creatinine (mg/mg)	0.22 ± 0.01	0.28 ± 0.02*
Citrate/Creatinine(mg/mg)	0.19 ± 0.05	0.15 ± 0.08*

BUN: blood urea nitrogen, PRA: plasma renin activity; UUN: urine urea nitrogen.

^*^P < 0.05 *vs.* WT mice.
