# Supplementary material for: Chronic Metabolic Acidosis Activates Renal Tubular Sodium Chloride Cotransporter through Angiotension II-dependent WNK4-SPAK Phosphorylation Pathway
Source: Sci Rep. 2016 Jan 5;6:18360. doi: 10.1038/srep18360 (PMC4700450; doi:10.1038/srep18360)

Supplementary Information

Chronic Metabolic Acidosis Activates Renal Tubular Sodium Chloride Cotransporter through Angiotension II-dependent WNK4-SPAK Phosphorylation Pathway

Yu-Wei Fang, MD[1], Sung-Sen Yang, MD, PhD, Chih-Jen Cheng, MD, PhD,

Min-Hua Tseng, MD, Hui-Min Hsu, BS, and Shih-Hua Lin, MD

Corresponding Author: Shih-Hua Lin, M.D.

Figure S1 Uncropped and unprocessed images of blots and gels in Figure 1A.

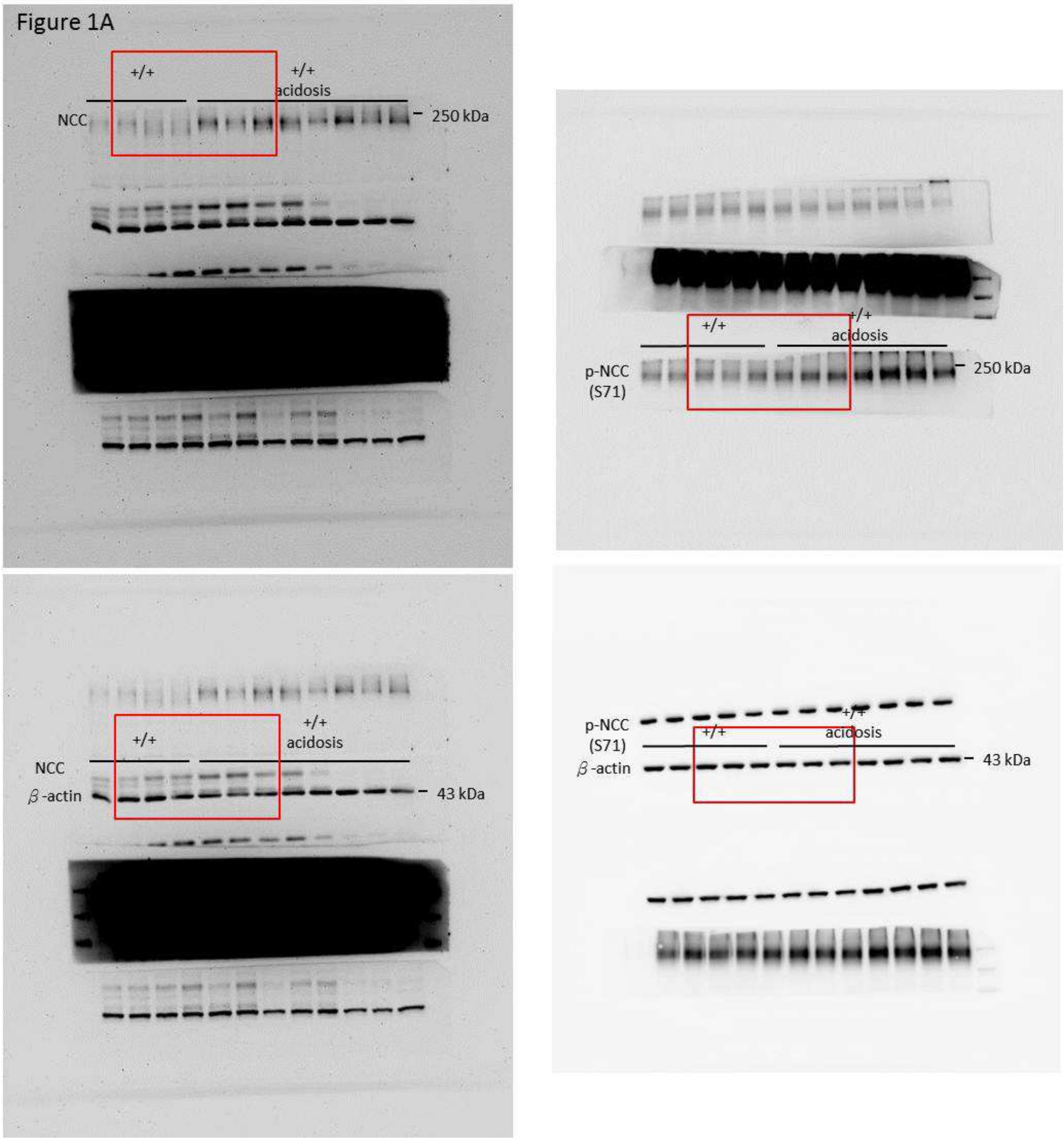

Figure S2 Uncropped and unprocessed images of blots and gels in Figure 1B

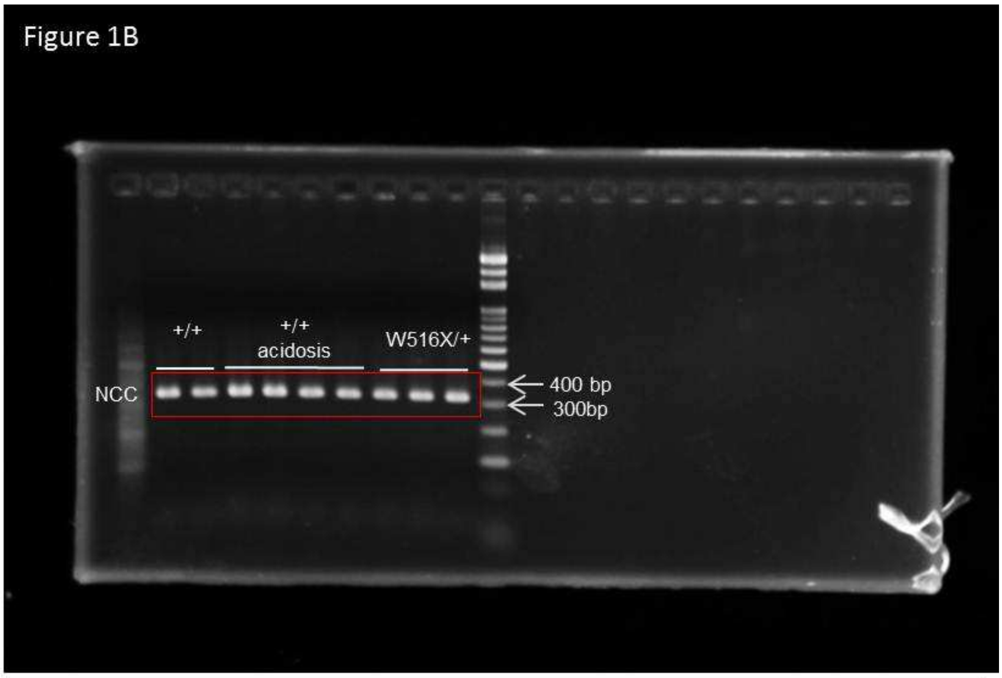

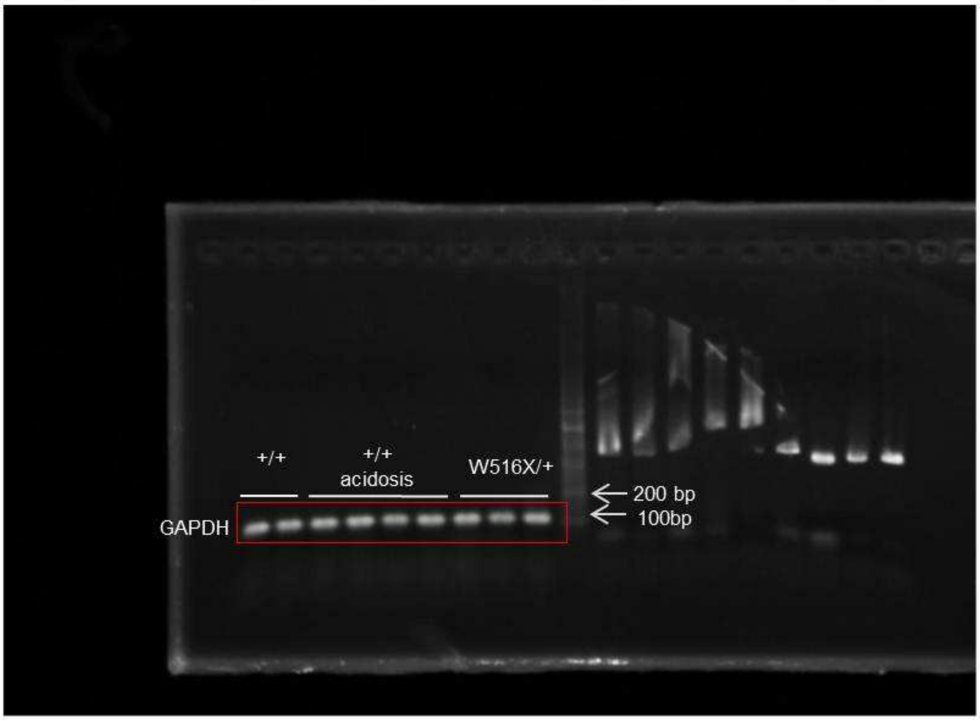

Figure S3 Uncropped and unprocessed images of blots and gels in Figure 2A

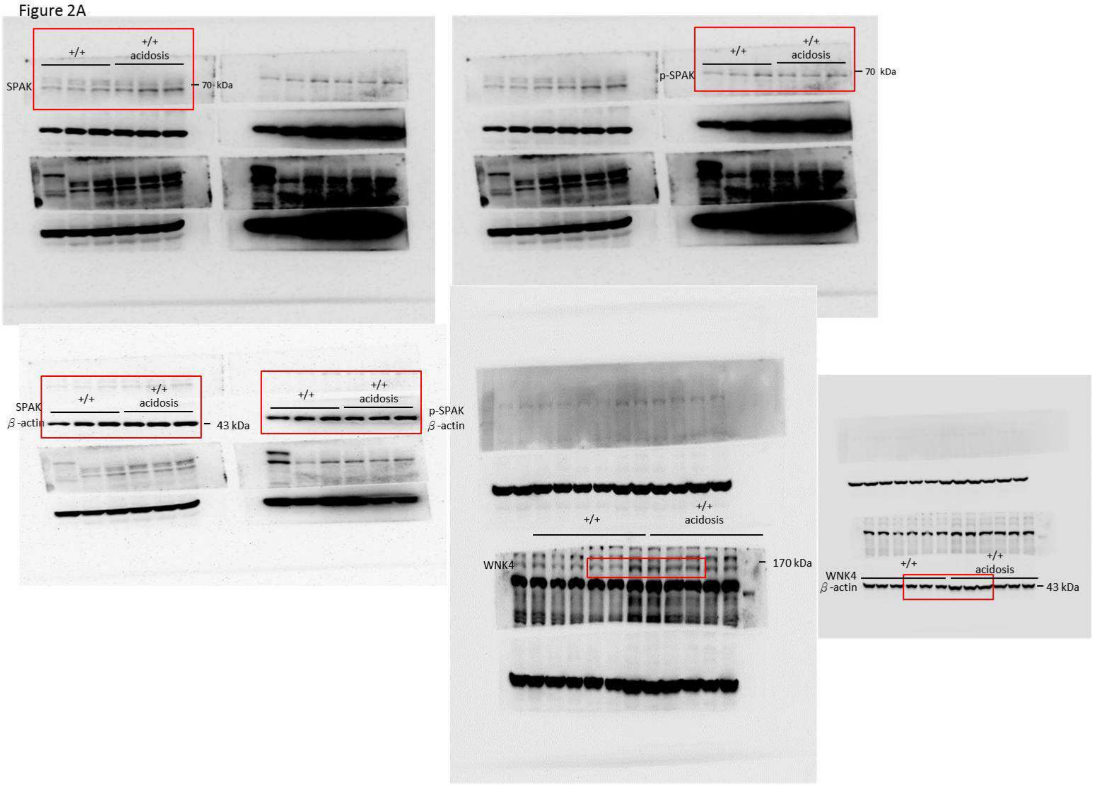

Figure S4 Uncropped and unprocessed images of blots and gels in Figure 3.

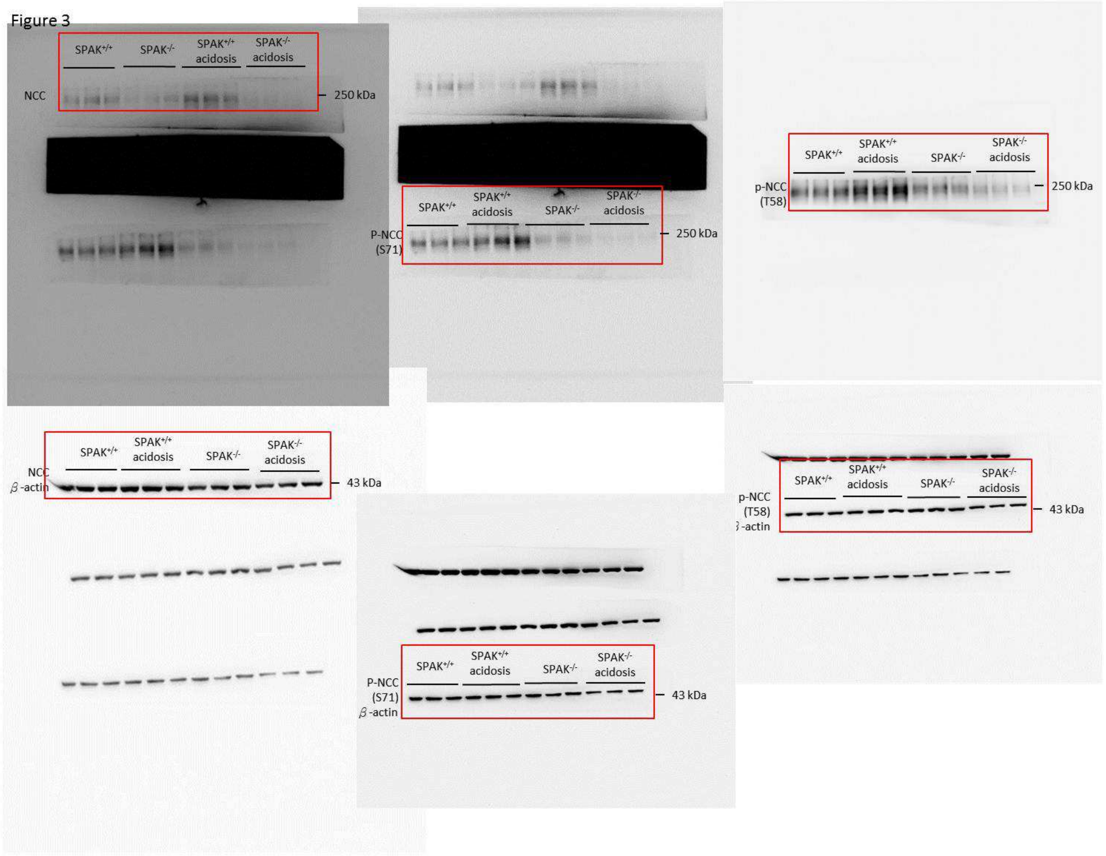

Figure S5 Uncropped and unprocessed images of blots and gels in Figure 4.

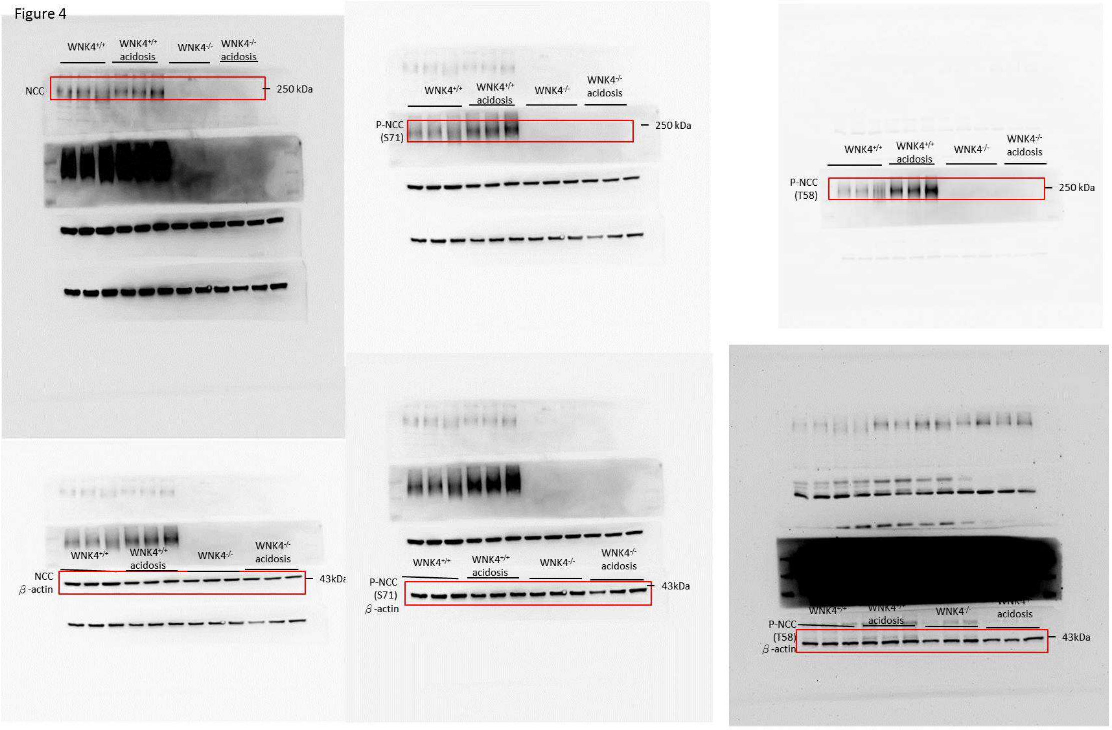

Figure S6 Uncropped and unprocessed images of blots and gels in Figure 5B.

Figure 5B

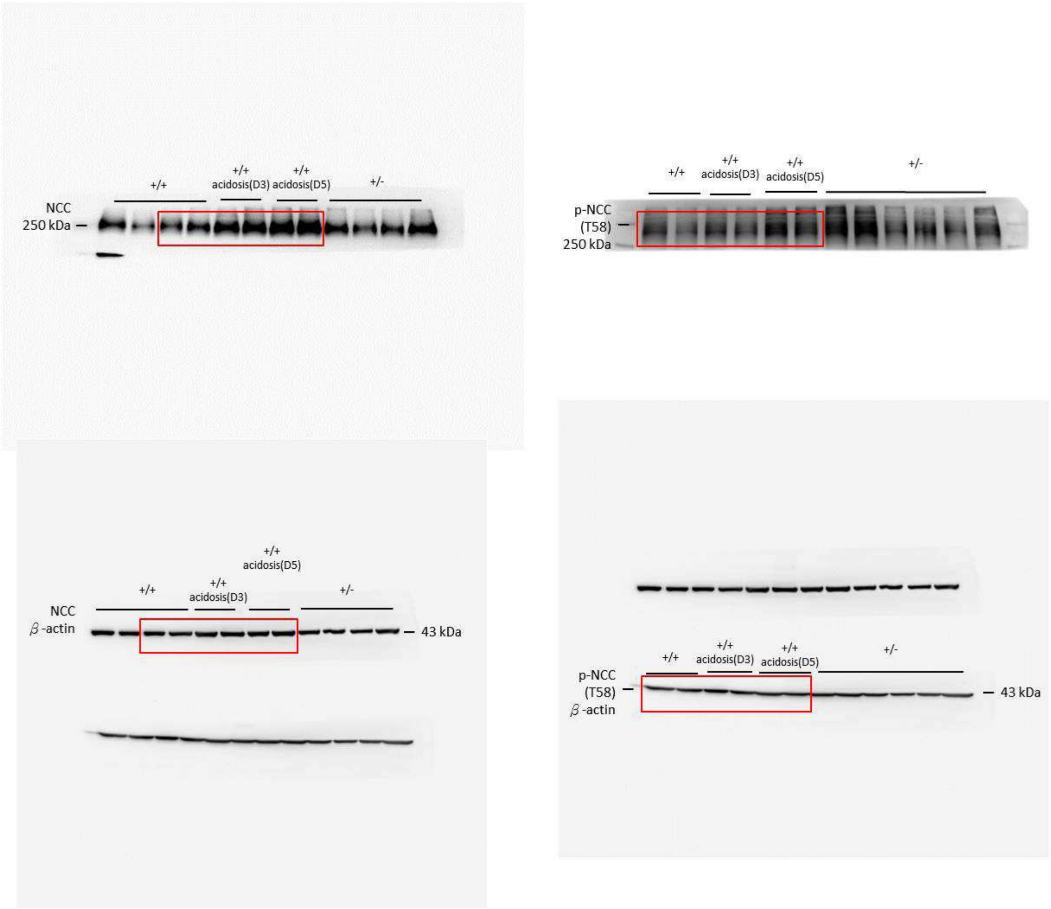

Figure S7 Uncropped and unprocessed images of blots and gels in Figure 6.

Figure 6

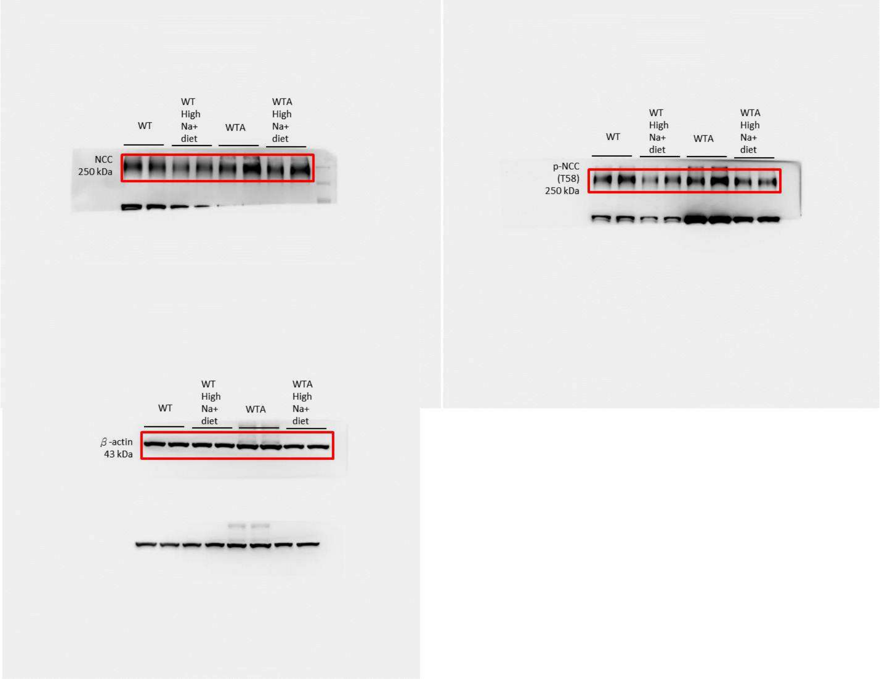

Figure S8 Uncropped and unprocessed images of blots and gels in Figure 7.

Figure 7

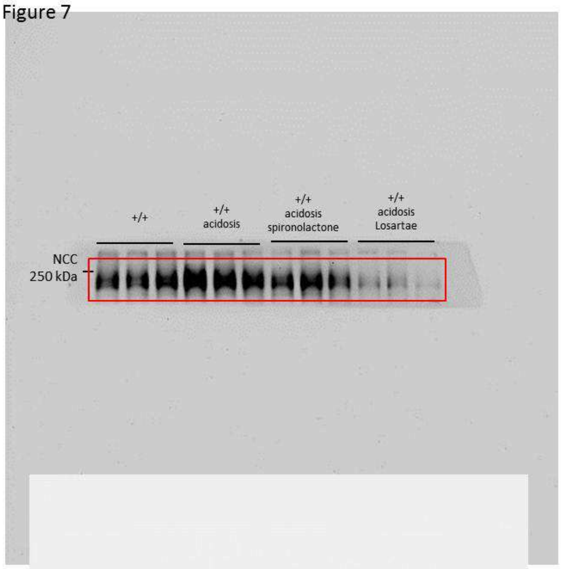

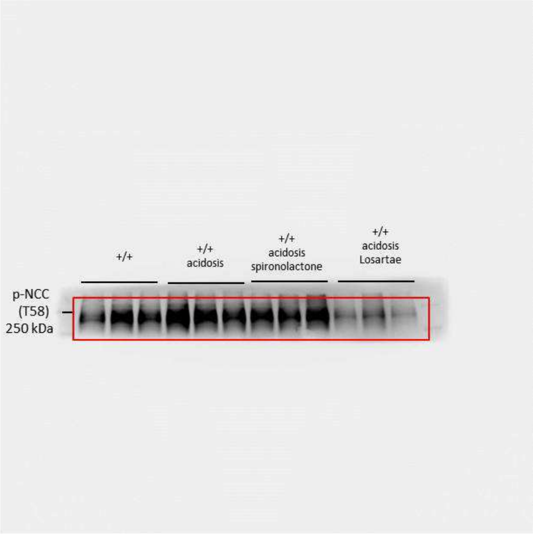

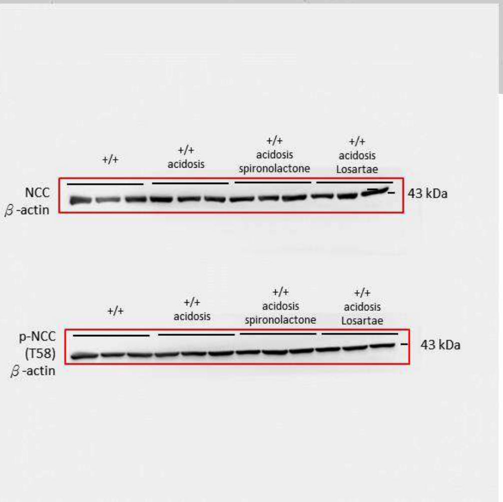

Supplement: Supplementary Information [file srep18360-s1.pdf]
